# Surface roughness effects on the frequency tuning performance of a nanoelectromechanical resonator

**DOI:** 10.1186/1556-276X-8-270

**Published:** 2013-06-07

**Authors:** Hyong Seo Yoon, Byeongho Park, Seong Chan Jun

**Affiliations:** 1 School of Mechanical Engineering, Yonsei University, Seoul, Korea

**Keywords:** Nanoelectromechanical resonator, Electrothermal tuning, Surface roughness

## Abstract

Electrothermal heating is one of radio frequency tuning method in nanoelectromechanical resonators with magnetomotive transduction. This study confirmed that the surface roughness of the nanoresonator affects the electrothermal tuning performance under moderate conditions at room temperature. The effect of surface roughness on electrothermal tuning is complicated and involves interactions of mechanical and electrical properties. In addition, the electrothermal damping varied depending on the nanoscale molecular solid structure. These factors affect the signal-to-noise ratio, the effective stress of the beam, and the quality Q-factor of the nanoresonator.

## Background

In nanoelectromechanical systems (NEMS), there are many demands such as a low power consumption, high signal-to-noise ratio (SNR), wide dynamic range, high critical value, and improved Q-factors. These issues are becoming increasingly important as resonators are developed for smaller devices in extremely sensitive applications [1] including sub-atto-Newton force sensing [2], single-spin detection [3], and quantum experiments [4,5]. Also, recent studies have reported the utilization of the photothermal effect to tune the frequency of a nanoresonator [6,7]. Tremendous efforts have been exerted to improve the Q-factor of electromechanical resonators over the past few decades, especially at smaller scales such as in the nanometer range. Operating a nanoresonator with a high Q-factor is the most crucial prerequisite for their practical application, and the stiffness, damping factor, noise, and dissipation factors are very important to maintain high Q-factor [8,9]. However, there are trade-offs with this approach. The diminishing device size effects provide higher sensitivity and frequency, whereas the Q-factor tends to decrease [10], and the resonance motion with higher Q-factor is easier to show nonlinear characteristic [11]. Comparatively, high-quality performance has been observed under extreme conditions such as low temperatures, high field forces, and high vacuums. Recently, many efforts have been made to apply this technology in practical conditions [10,12,13]. However, it is difficult to maintain the Q-factor of the nanoelectromechanical resonator at a high level for radio frequency resonating because of mechanical and electrical damping effects experienced under moderate operating conditions.

Moreover, in the nanoscale structure, the surface roughness can be a significant issue for electron and phonon transmission or scattering [14,15] since the surface-to-volume ratio increases. Electron and phonon scattering in the atomic solid state of the resonator is dominant with inter-atomic or inter-boundary structural changes due to thermally enhanced phonon–electron interactions by the electrothermal power. Therefore, in this study, Q-factor issues associated with the surface roughness of the resonator were analyzed under moderate conditions while performing frequency tuning.

After the nanomechanical resonator showed successful operation of the radio frequency (RF) resonance, deepening research topics of various working conditions have been investigated including frequency tuning [16], controlling the nonlinearity of resonating [17], and chemical vapor sensing [12,18]. In our study, a doubly clamped nanoscale resonator using electromagnetomotive transduction was operated under a moderate vacuum (about 1 Torr) at room temperature with a B field of 0.9 T. Also, an RF tuning method was adopted in a magnetomotive transduction operation. It was previously demonstrated that linear tuning with an input power appears to be feasible at the application level with a low electrothermal power consumption of only a few microwatts [16]. In addition to resonance frequency tuning, the Q-factor must be analyzed in order to maintain quality performance without degradation under moderate conditions.

The dissipation and damping effects which limit the performance of beam resonance are mainly induced from various causes such as physical defects from the beam structure, thermal elastic damping from the surroundings, clamping losses from supporting clamps on the substrate, signal loss due to electrical feedthrough, and phonon–electron couplings. Therefore, in our study, much effort was made to carefully construct and test the experimental conditions in order to minimize the dissipation factors except for the surface roughness of the resonator.

## Methods

SiC provides superior material properties for high-frequency applications due to its high stiffness and low density, as well as its good tunability due to its higher thermal conductivity than other NEMS materials such as silicon and silicon nitride. Even though it has excellent mechanical properties including a high Young's modulus and low density, a drawback of SiC is its low electric conductivity. In this work, Al layers were applied to the surface of SiC to improve its conductance. This hybrid layer structure (Al/SiC) is a main loss factor but still results in comparable performance to other materials, which must be produced via careful fabrication processes.

**Figure 1:**
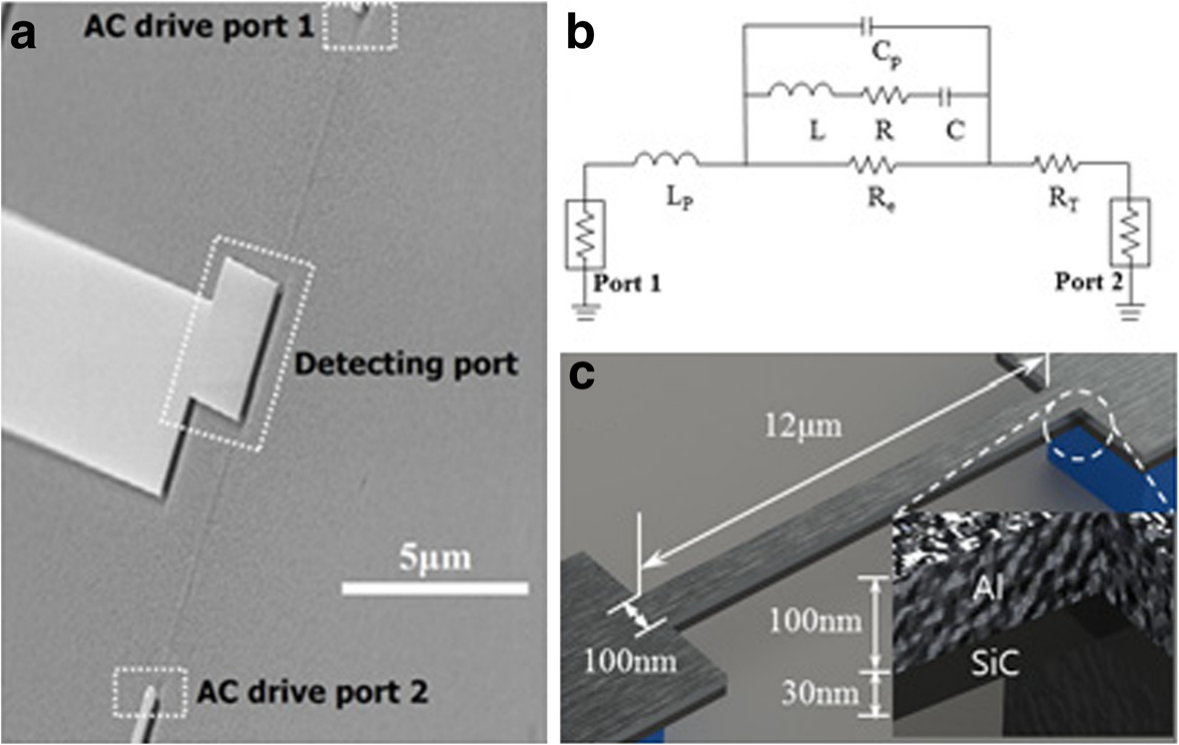
**SEM images of the experimental setup.** (**a**) Experimental setup of resonance detection using a balanced bridge. (**b**) The equivalent circuit model. (**c**) Schematic image of the beam with the geometric detail.

Scanning electron microscopy (SEM) images of the experimental apparatus and a fully suspended beam are presented in Figure 1a. The electrical equivalent circuit model is shown in Figure 1b. R, L, and C are the resistance, inductance, and capacitance, respectively, to model the fundamental resonance response of the beam resonator. The further electron and phonon scattering due to the rough surface will induce higher resistance, R, and more damping. R_e_ is the equivalent resistance due to the substrate and other environment including the energy loss or thermal dissipation. Also, there are parasitic capacitance and inductance, C_p_ and L_p_, from the beam structure or metal pad and read out. R_T_, the thermal resistance represents the energy dissipation due to the DC thermal voltage applied for the frequency tuning. The composite nanoresonators are 12-μm long, 100-nm wide, and 130-nm (3C-SiC 30 nm, Al 100 nm) thick as shown in Figure 1c. Ultrathin single crystal 3C-SiC films were grown on a silicon wafer by a heteroepitaxial atmospheric pressure chemical vapor deposition process in which SiH_4_ and C_3_H_8_ were used as precursors [19] followed by deposition of the Al layer. In order to analyze the surface roughness effects of the resonator, careful fabrication is essential and mandatory. It is crucial to determine the final surface roughness of the Al layer, which is the topmost layer in the resonator, even though the final roughness of the resonator surface is determined by both the 3C-SiC and Al fabrication conditions. The initial deposition conditions are extremely important for stacking the atomic arrangement, which mostly determines the final roughness of the resonator. We gradually changed the deposition rate of Al from a very low level to moderate conditions for each sample. The initial deposition rate of less than 0.2 nm/min was gradually increased to 1 nm/min. Therefore, the final surface roughness of the nanoresonator varied widely depending on the deposition rate of Al, as shown in Table 1. Here, a slow deposition rate yields a low roughness as well as a formable bond between SiC and metal, which results in a high initial Q-factor. The composite layered film is patterned by e-beam lithography after the application of a PMMA resist (495 KDa). Lift-off follows, and then, the DRIE is implemented to etch away the Si substrate applying predefined parameters in order to fully suspend it without any residues. The fabrication process parameters such as the deposition rates of the materials and working temperature strongly affect the stress distributions of nanoresonators as well as the quality factor. Controlling these factors can improve the reliability and sensitivity of the nanoresonator.

**Table 1:** The surface roughness of the resonators and their standard deviation values

**Factor**	**Resonator**			
	**R #1**	**R #2**	**R #3**	**R #4**
Roughness (nm)	11.2	28.8	0.9	2.4
SD (nm)	5.2	17.3	0.7	1.5

**Figure 2:**
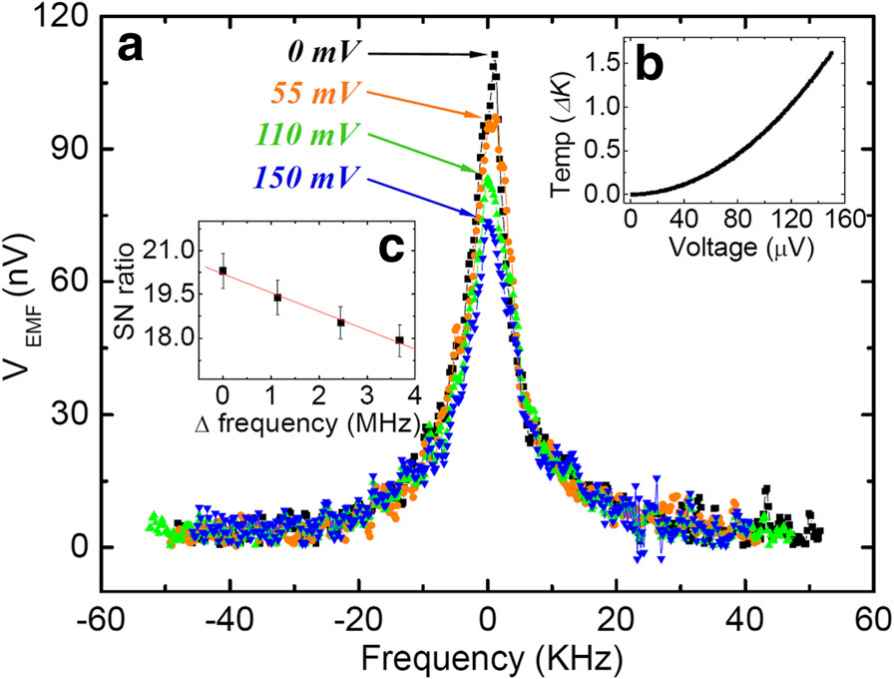
**Resonance properties of frequency, temperature changes from electrothermal voltage, and signal-to-noise ratio of resonant frequency.** (**a**) The resonance properties of the electrothermally tuned frequency at various voltages. (**b**) The temperature changes resulting from the electrothermal voltage. (**c**) The signal-to-noise ratio as a function of the resonant frequency.

In the setup, the nanoscale doubly clamped resonator is loaded onto a printed circuit board (PCB) and connected to a moderate vacuum chamber at room temperature, which is affected vertically by a magnetic field (0.9 T). An analog current drive of at least a few tens of microvolts is sent through two ports of the PCB board, which are connected to the beam ends. The electromagnetic field voltage, which is induced by the Lorentzian excitation principles of the resonators, is detected by an amplifier-powered readout port connected to a network analyzer (Agilent E5071C, Agilent Technologies, Inc., Santa Clara, CA, USA), as shown in Figure 2a.

## Results and discussion

The resonant frequency of a doubly clamped beam under thermal stress induced by electrothermal power can be represented as follows [13]:

(1) $$\omega_{0}^{2}={\left(\frac{2\pi }{L}\right)}^{4}\left[\frac{\mathrm{EI}}{3\mathrm{\rho A}}\left(1+\frac{T_{f}}{\mathrm{EI}}{\left(\frac{L}{2\pi }\right)}^{2}\right)\right]$$

where *A* is the beam cross-sectional area, *L* is the length of the beam, *ρ* is the effective density of the beam, *E* is the effective Young's modulus, and *T*_*f*_ is the beam tension which is proportional to the temperature change of the beam as below:

(2) $$\Delta T_{f}=\mathrm{E\alpha}\left(\mathrm{\Delta T}\right)A$$

As presented in the equation, the beam stress is closely related to the resonance frequency and the Q-factor is also affected by changes of the beam stress via electrothermal stress due to critical parameters such as the thermal time constants and thermal conductivity. The resonant frequency is subject to variations despite the identical geometries and materials of the beams because of the different effective beam stresses. Predicting the resonant frequency is difficult due to the stress distributions over the beam structure, which is primarily caused by the different layer deposition conditions and the resulting molecular compositions.

**Figure 3:**
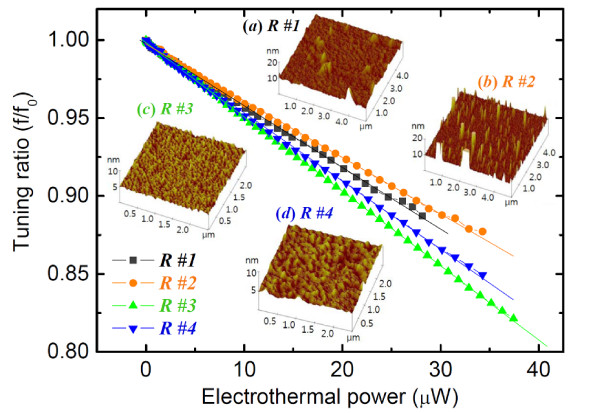
**Frequency tuning performance as a function of surface roughness of nanobeam.** Observed in AFM image of surface morphology of Al-SiC. The surface roughness is a key parameter for the resonant frequency and tuning performance. The average roughness of the (**a**) R#1, (**b**) R#2, (**c**) R#3, and (**d**) R#4 samples varies from less than a nanometer to 30 nm.

Figure 2a shows comparisons of the transition of resonance peaks as the tuning power changes, which induces the temperature increment of the doubly clamped beam, as shown in Figure 2b, and generates different Q-factors. The amplitude of the resonance oscillations decreases with increasing tuning power. Even though the resonance peaks shifted from 111.35 nV at a DC voltage of zero to 73.62 nV at 150 mV, the nonlinearity operation of the beam is recovered for linear operation via DC tuning. During the period of time in which the Q-factor decreases and the frequency tuning increases, the SNR is also reduced, as shown in Figure 2c. While the tuning power is supplied for the frequency shift, it may allow the external environment to couple with the softened beam structure due to Joule's heating. resonant frequency is tuned downward as the tuning voltage is applied, as shown in Figure 3. When operating in the range of the radio frequency resonance with a magnetomotive transduction technique, the tuning ratio is varied by the Lorentzian force. Furthermore, these effects depend on the surface roughness of the resonator. The device with a smaller roughness, as determined by the atomic force microscope (AFM) measurements shown in the inset of Figure 3, was tuned more easily. The effect of the surface roughness complicates the loss of resonating performance and also makes the performance more difficult to predict. These phenomena cause discrepancies and deviations from the theoretical predictions.

**Figure 4:**
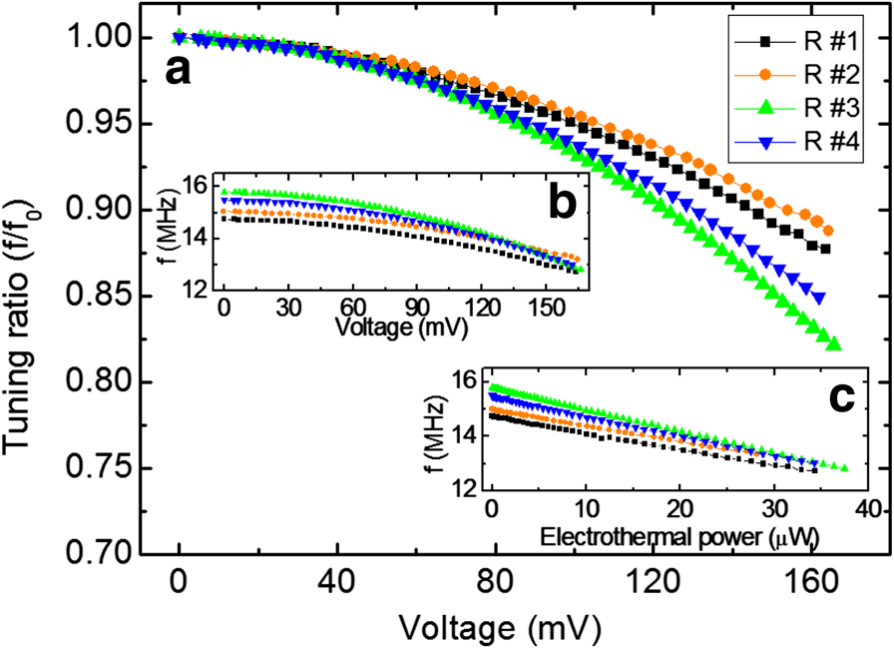
**Electrothermal damping effects on a nanoelectromechanical resonator.** (**a**) Tuning ratio from the original resonance frequency in terms of the tuning voltage. (**b**) Actual tuned frequency based on the tuning power. (**c**) The variation of the resonance frequency resulting from tuning the voltage.

The results also demonstrate how electrothermal-powered frequency tuning is affected by the surface conditions of the beam, which results in the determination of the tuning ratio's stability and linearity, based on the input power. Figures 3 and 4 show that the beam with the smallest roughness can obtain the highest tuning ratio from the original resonant frequency. With the same amount of thermal power input, the tuning ratio decreases as the surface roughness increases. The dissipation prevails more on a rougher surface due to electron scattering, energy loss, and unequal or non-uniform electrothermal heating.

The initial resonant frequencies, which are different for each beam, do not affect the frequency tuning ratio, as shown in Figure 4b,c. Furthermore, the stress of the beam is closely correlated to the quality factor during frequency tuning with the nanoelectromechanical resonator, which has a low surface roughness and a well-suspended beam. Actually, the amount of stress or changes of the Q-factor are caused by increased external force due to surface roughness [20].

**Figure 5:**
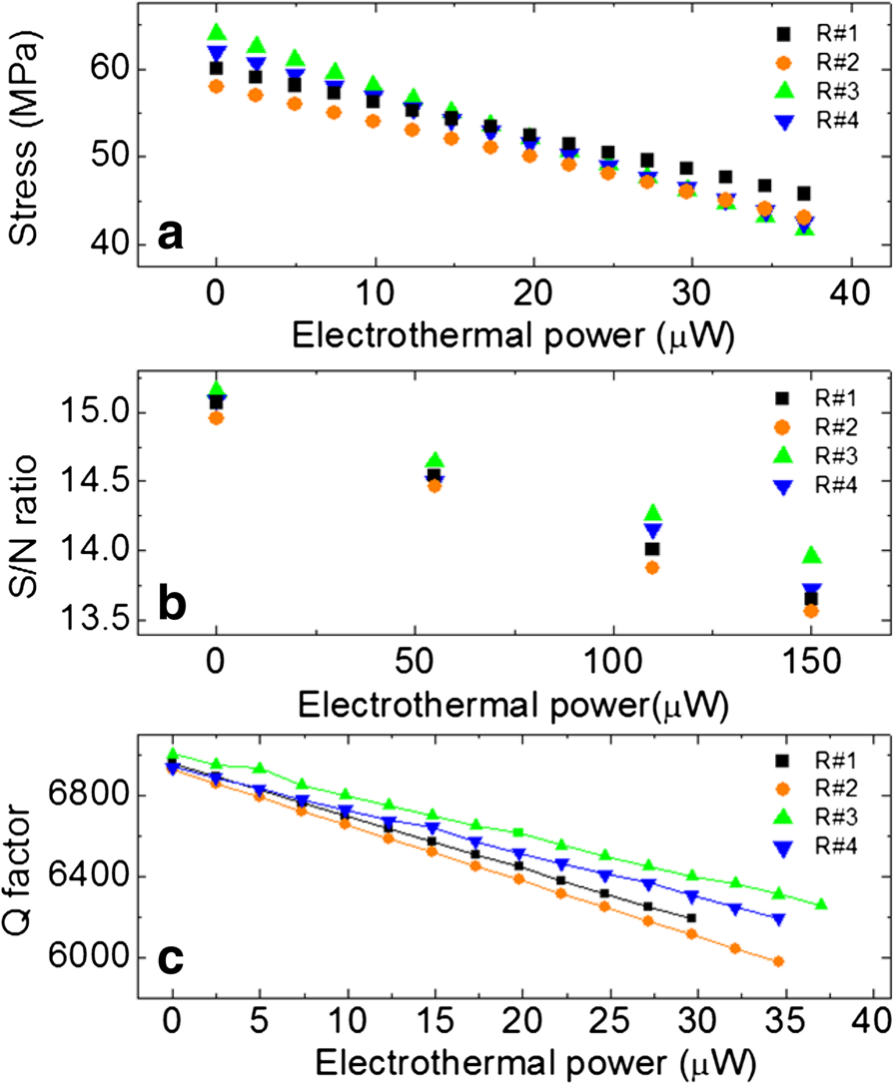
**Results from electrothermal frequency tuning.** (**a**) stress distribution, (**b**) signal-to-noise ratio, and (**c**) Q-factor as a function of the surface roughness.

Figure 5a shows the effective stress of the resonator transformed by the tuning power, which suggests a correlation between the effective stress and quality factor. The signal-to-noise ratio at various surface roughnesses is shown in Figure 5b. It is presumed that the finest surface results in the highest SNR, but this is not clearly distinguishable. However, the SNRs of the #1 and #2 resonators with rougher surfaces were lower. The quality factors were evaluated while the frequency tuning operation was performed, as presented in Figure 5c. With regards the Q-factor during electrothermal tuning, initially, the finest surface of R#3 had a slightly higher Q-factor than the other samples and the degradation of Q-factor with electrothermal effects was also relatively lower than with a rougher surface of the resonator. The Q-factors decreased slowly as the thermal power was increased from 0 to 150 mV, while the resonance frequency decreased linearly. As the resonant frequency is tuned, the Q-factor decreases due to scattering and noise effects, which are mostly affected by the physical properties of the nanoscale beam because Joule's heating from the electrothermal power reduces the strength of the beam, which further causes a transition of the Q-factor. In order to maintain high resonator performance, the Q-factors should be kept as high as possible, especially in room temperature magnetomotive transduction where there are many sources of loss.

The tuning performance is primarily decided by the effective beam stress of resonator, which controls not only the resonant frequency but also the resonant properties of the Q-factor, dynamic range, and SNR. The beam stress distributions may be critically determined by the surface roughness, especially at the nanoscale since the surface roughness suggests not only the defects on the surface but also the intermolecular binding condition beneath the surface in the very thin structure. There are two main issues regarding the effects of the surface roughness on the electrothermal tuning performance. One is that the electric conductivity and thermal conductivity are closely related to the tuning performance, which is induced from decreasing electron and phonon transfer through a conducting layer. The other factor is the mechanical stress distribution with thermal stress provided by the electrothermal power. Intermolecular expansion or subtraction interaction occur either regularly or irregularly, which is decided by isotropic or anisotropic molecular bindings. These mostly depend on the surface roughness and sub-layer structure, which affect the boundary between the SiC and Al composite layers. The Al layer tends to be affected by tensile stress whereas SiC is dominated by compression stress while undergoing electrothermal tuning. Those opposite stress distributions from composite layers, especially at the boundary layer, make the tuning effects clearly different from other various molecular structures.

Because the thermal damping effects on mechanical resonant motions over a megahertz resonant range are not trivial and many complicated effects exist regarding the thermal expansion among intermolecular bonding, the thermal stress over tight-binding solid structures is increased. These effects are mainly concentrated on the top metal layer of the composite resonator beam with a thickness of a few tens of nanometers, which is small enough to be sensitive to intermolecular stress changes induced by thermal stress. The nanoscale mechanical structure of a beam atomically deposited by chemical vapor deposition is highly related to the top layer surface roughness. From another point of view, the mechanical motion is primarily determined by a balanced weight distribution, especially in high frequency motion. Various unbalanced weight bumps distributed on the top of the surface increase the surface roughness, which strongly affects the resonant motions, contributing to Q-factor degradation. In the case of a nanoscaled beam, the roughness effects play a non-trivial role in RF motion.

## Conclusions

We demonstrated that as the size of the NEMS beam decreases, the effect related to the beam surface roughness becomes the dominant characteristic due to a large surface-to-volume ratio. The frequency tuning performance was improved with less electrothermal power consumption by improving the surface roughness of the Al-SiC nanobeam. The surface roughness should be controlled in order to minimize the loss of the RF tuning performance. The surface roughness effects are related to not only electromechanical resonance performance but also to electrothermal conductance and dissipation, which are emphasized more in nanoscaled devices because electron and phonon interactions are complicated with scattering issues.

## Competing interests

The authors declare that they have no competing interests.

## Authors’ contributions

HY carried out the resonator operation and drafted the manuscript. BP carried out the resonator fabrication and AFM measurement. SJ supervised the experiment and conceived of the study. All authors read and approved the final manuscript.
